# Epidemiology and Management of Ankle Fractures Prior to, During, and Following the COVID-19 Pandemic in an Italian Tertiary Hospital

**DOI:** 10.3390/medicina61081439

**Published:** 2025-08-10

**Authors:** Gianluca Testa, Francesco Leonforte, Marco Sapienza, Martina Ilardo, Stefania Garozzo, Maria Agata Musumeci, Michela Marchetti, Andrea Vescio, Antonio Mistretta, Vito Pavone

**Affiliations:** 1 Department of General Surgery and Medical Surgical Specialties, Section of Orthopaedics and Traumatology, Policlinico Rodolico-San Marco, University of Catania, 95123 Catania, Italy; gianpavel@hotmail.com (G.T.); stefaniagarozzo2@gmail.com (S.G.); musumeci.maria.mm@gmail.com (M.A.M.); michelamarchetti@hotmail.com (M.M.); vitopavone@hotmail.com (V.P.); 2 Department of Integrated Hygiene, Organizational, and Service Activities (Structural Department), Health Management, University Hospital Policlinic “G. Rodolico-San Marco”, 95123 Catania, Italy; francesco.leonforte@policlinico.unict.it; 3 Department of Life Science, Health, and Health Professions, Link Campus University, 00165 Rome, Italy; andreavescio88@gmail.com; 4 Department of Orthopaedic and Trauma Surgery, “Mater Domini” University Hospital, “Magna Græcia” University, 88100 Catanzaro, Italy; 5 Hygiene Unit, Department of Medical and Surgical Sciences and Advanced Technologies, University of Catania, 95124 Catania, Italy; anmistre@yahoo.it

**Keywords:** ankle fractures, COVID-19 pandemic, trimalleolar fractures, surgical treatment trends, epidemiology

## Abstract

*Background and Objectives*: Ankle fractures represent one of the most common injuries to the lower limb, particularly impacting women and the elderly. The coronavirus disease 2019 (COVID-19) pandemic greatly disrupted both the incidence of these fractures and their treatment patterns globally. This retrospective epidemiological study analyzed 1010 cases of ankle fractures treated at the Orthopedics Department of Policlinico University Hospital in Catania from 2018 to 2023. The study aimed to evaluate trends in incidence, patient demographics, fracture types, treatment modalities, and hospital stay across the pre-COVID-19, COVID-19, and post-COVID-19 periods. *Materials and Methods*: A retrospective observational study was conducted including all patients diagnosed with ankle fractures from 1 January 2018 to 31 December 2023. Data were collected from hospital medical records using ICD-9-CM codes and radiographic classification systems (Danis–Weber, Lauge-Hansen, and AO/OTA). Variables analyzed included demographics, fracture type and side, treatment modality, and hospitalization details. Statistical analyses were performed using t-tests, chi-square tests, and linear regression, with significance set at *p* < 0.05. *Results*: In 2020, there was a 31.7% decrease in fracture incidence. Although overall fracture rates rebounded after COVID-19, they did not reach pre-pandemic levels. During the pandemic, trimalleolar fractures increased significantly, occurring more frequently in older women, likely due to bone fragility. The rate of surgical treatments rose during and after the pandemic, with a distinct shift from ORIF to external fixation. Hospital stays were longer, especially for patients with cardiovascular risk factors. *Conclusions*: The pandemic significantly altered the epidemiology, treatment strategies, and outcomes of ankle fractures. These findings highlight the necessity for adaptable care models and preventive strategies, particularly for vulnerable populations such as older women.

## 1. Introduction

Ankle fractures are among the most common fractures in adults, with an incidence rate of 174 per 100,000 person-years [1]. They account for approximately 10% of all adult fractures, ranking second only to hip fractures among lower limb injuries. The incidence rates range from 71 to 187 per 100,000 person-years and are predominantly observed in adults under the age of 60. However, there is an increased fracture risk in postmenopausal women [2,3]. Studies indicate a significant rise in incidence among females, particularly those over 65 years of age. Notably, the age-adjusted rates in Finland doubled from 1970 to 2000 [4].

The incidence of ankle fractures in women typically peaks between the ages of 60 and 70, after which it either stabilizes or declines. These fractures, frequently resulting from low-energy trauma, become more common with advancing age and predominantly affect women. There is a noticeable peak in incidence for women between the ages of 30 and 60, whereas men experience these fractures more uniformly across different ages [5]. Among adolescents, fracture rates are highest, with males experiencing 9.5 fractures per 10,000 person-years and females experiencing 5.5. In subsequent decades, the incidence for males declines. In contrast, women demonstrate a bimodal distribution, with a secondary peak occurring after age 80. This later peak is likely associated with osteoporosis and reduced bone density, commonly observed in older age [6,7,8].

In a decade-long study involving over 50,000 cases, most ankle fractures in women were caused by low-energy trauma. In contrast, men more frequently sustained fractures from high-energy trauma. These findings suggest that ankle fractures in elderly women, linked to factors such as reduced muscle mass and increased body mass index (BMI), particularly from simple falls, may be considered osteoporotic [9,10]. Seasonal conditions, such as snow and ice, significantly influence fracture rates, which peak from November to March. This pattern is consistent with studies from Nordic countries that report similar seasonal variations in humerus, femur, and ankle fractures [11].

The management of ankle fractures typically involves interventions such as surgery, immobilization, and rehabilitation. The coronavirus disease 2019 (COVID-19) pandemic posed significant challenges to fracture treatment, leading to a notable decrease in the incidence of ankle fractures. This decline was likely due to government restrictions, reduced physical activity, and patients’ reluctance to visit hospitals out of fear of COVID-19 exposure [12,13]. The reduction was particularly pronounced during the first 30 days of the pandemic, with higher adherence to restrictions noted among women and individuals over 70 [14].

The pandemic adversely affected ankle fracture management outcomes, leading to increased surgical delays, extended hospital stays, and higher rates of complications such as pneumonia, deep vein thrombosis, and sepsis. Mortality rates among fracture patients were reported to be up to twice as high as pre-pandemic levels. This increase was particularly pronounced among elderly patients, whose physical frailty and pre-existing comorbidities placed them at a higher risk of adverse outcomes [12,15].

During the pandemic, injury patterns changed significantly. Sports-related trauma dropped markedly due to the suspension of sports and outdoor activities, while motor vehicle accidents constituted the largest percentage of trauma cases before COVID-19. In contrast, work-related injuries and high-energy falls increased as people spent more time at home, leading to a rise in domestic accidents. Among the elderly, falls from lower heights became common, resulting in an increase in fragility fractures. Injuries sustained in public spaces, schools, daycare centers, residential care facilities, and sports areas decreased, while injuries at home increased nearly fourfold. This increase is likely attributable to extended periods spent indoors [15].

During the pandemic, orthopedic practice routines adapted by postponing many elective surgeries and utilizing telemedicine whenever feasible [16,17,18,19]. While urgent fracture care remained ongoing, there was a notable shift in both injury mechanisms and the timing of patient presentations. Some patients opted for telemedicine consultations, a trend that grew following the implementation of shelter-in-place mandates. Consequently, the pandemic resulted in a significant shift in the types of injuries managed in orthopedic foot and ankle clinics, mirroring changes in activity levels and patients’ reluctance to seek in-person medical care for non-urgent injuries [20,21].

The primary objectives of this study are to analyze the epidemiological trends of ankle fractures across three distinct periods: pre-COVID-19, during COVID-19, and post-COVID-19. By examining variations in incidence, treatment methods, and patient demographics, the study seeks to provide insights into how these factors influence fracture patterns and management strategies.

## 2. Materials and Methods

### 2.1. Study Design

This study is a retrospective observational epidemiological analysis focusing on ankle fractures treated at the Orthopedics Department of Policlinico University Hospital, “G. Rodolico—San Marco,” from 1 January 2018 to 31 December 2023. The primary objective was to evaluate the epidemiological trends of ankle fractures across three distinct periods: pre-COVID-19 (1 January 2018–31 December 2019), during COVID-19 (1 January 2020–31 December 2021), and post-COVID-19 (1 January 2022–31 December 2023).

### 2.2. Inclusion and Exclusion Criteria

Inclusion criteria specify that patients of any age diagnosed with an ankle fracture requiring orthopedic treatment between 1 January 2018 and 31 December 2023 are eligible for the study. The exclusion criteria disqualify patients with additional fractures involving bones outside the ankle.

### 2.3. Data Collection

Data for this study were retrieved from the hospital’s medical records database using the International Classification of Diseases, 9th Revision–Clinical Modification (ICD-9-CM) codes [22]. The dataset included all patients admitted and treated for ankle fractures in the Orthopedic Unit during the study period. Information obtained from the electronic hospital admission system included demographic details (sex and age), diagnostic codes, comorbidities, length of hospital stay, and other relevant clinical variables. Radiographic data were accessed through the Carestream^®^ imaging system, a certified medical imaging archive adopted by the institution. Fractures were assessed and classified according to the Danis–Weber, Lauge-Hansen, and AO/OTA classification systems [23]. Radiographic evaluations were independently conducted by two orthopedic surgeons experienced in trauma care. Any discrepancies between reviewers were resolved by consensus through joint review. To ensure accuracy and consistency, data entries were cross-verified by a third reviewer. All information was anonymized prior to analysis in compliance with institutional data protection standards. The variables collected included demographics (sex and age), fracture details (type of fracture and affected side), clinical data (date of injury and medical history), and treatment details (type of treatment (surgical or non-surgical), length of hospital stay, date of surgery, and type of fixation used (if applicable)).

### 2.4. Statistical Analysis

Descriptive statistics for categorical data were presented as counts and proportions (%). Continuous data were summarized using the mean and standard deviation (SD), as well as the median and range.

Statistical comparisons employed various methods. T-tests evaluated differences in continuous variables, such as the length of hospital stay. Linear regression analyzed the relationship between fracture type and variables like sex or gender. Chi-square tests explored associations between categorical variables, including fracture type and treatment method. Statistical significance was defined as *p* < 0.05. To identify trends, we cross-referenced data on the type of malleolus involved with the management approach, highlighting preferences for surgical versus non-surgical treatment.

## 3. Results

### 3.1. Study Sample

The study included 1010 patients with malleolar fractures, comprising 534 women (52.87%) and 476 men (47.13%), yielding a male-to-female ratio (M:F) of 1:1.14. The mean age of the sample was 47.80 ± 20.37 years, with a range from 4 to 93 years.

The distribution of fractures over the years is as follows: 172 in 2018, 224 in 2019, 153 in 2020, 149 in 2021, 168 in 2022, and 144 in 2023. When categorizing the fractures into three reporting periods, we identified 396 fractures during the pre-COVID-19 period (2018–2019), 302 fractures during the COVID-19 period (2020–2021), and 312 fractures during the post-COVID-19 period (2022–2023) (Table 1).

The average incidence of these fractures is 56 ± 9.78 per 100,000 population per year, ranging from 50 to 75, during the period from 2018 to 2023 (Table 2).

### 3.2. Gender and Age

For men, the average annual incidence is 54 ± 10.91 (range: 44–69) per 100,000 population per year. For women, it is 57 ± 10.37, ranging from 48 to 77 per 100,000 population per year. This gender distribution is consistent with the described trend and does not exhibit significant differences across the compared time periods (*p* > 0.05) (Table 3).

**Figure 1:**
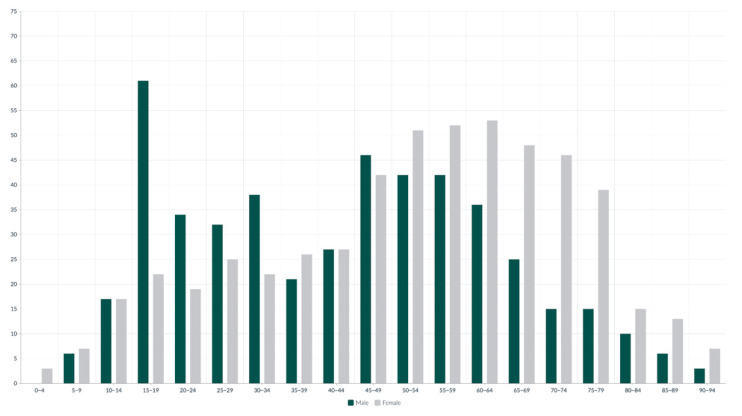
Number of patients with ankle fractures in 5-year age groups divided into male and female.

The study found that men were generally younger at the time of injury, with a mean age of 44.66 ± 19.64 years (range: 6–92 years), compared to women, whose mean age was higher at 52.30 ± 19.90 years (range: 4–93 years) (Figure 1).

**Figure 2:**
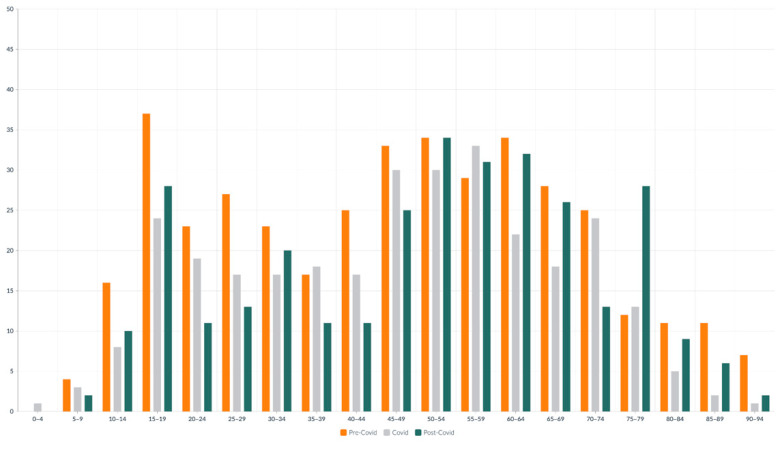
Number of patients with ankle fracture in 5-year age group distributed by period.

In the 15–19 age group, fractures predominantly occur in males, who account for 73.49% (61 patients) of the total fractures within this group. In contrast, beginning with the 50–54 age group, there is a gradual decline in fracture incidence among males, accompanied by a steady increase among females. This trend progresses until it peaks in the 60–64 age group, where females represent 59.55% (53 patients) of total fractures. In the younger age groups, the 0–4 age group shows a fracture incidence of 0.10% (1 patient), and the 5–9 age group accounts for 0.89% (9 patients). Similarly, in the older age groups, the 85–89 age group accounts for 1.88% (19 patients), and the 90–94 age group represents 0.99% (10 patients) of the total fractures (Figure 2).

### 3.3. Type of Fractures and Affected Side

**Figure 3:**
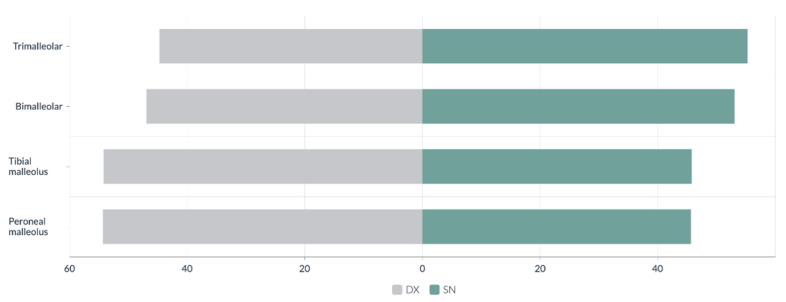
Distribution by side based on the number and type of malleolus involved.

The identified fracture types include 130 trimalleolar, 144 bimalleolar, 147 tibial malleolus, and 589 peroneal malleolus. Closed fractures comprised 991 cases (98.12%), while open fractures accounted for 19 cases (1.88%). The right side was involved in 507 patients (51.87%), while the left side was affected in 474 patients (48.13%) (Figure 3).

Trimalleolar fractures are more common in older females, with 63.85% (83 cases) occurring in the 50–79 age group. Conversely, tibial fractures are more prevalent among younger males, with 18.94% (25 cases) found in the 20–29 age group (Table 4).

### 3.4. Seasonality

**Figure 4:**
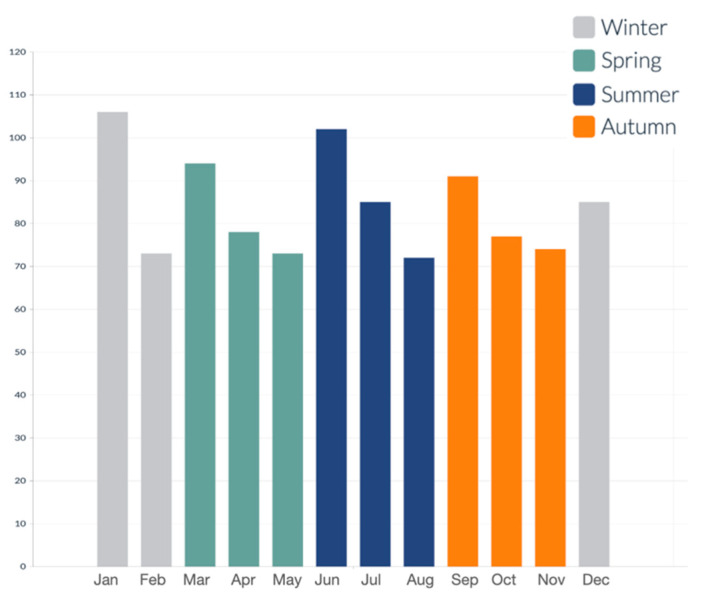
Number of ankle fractures distributed by season.

An analysis of seasonality reveals distinct differences in the monthly distribution of incidents throughout the year. Specifically, 26.24% (265) of the total incidents occurred during winter, 24.26% (245) in spring, 25.75% (260) in summer, and 23.76% (240) in autumn. This distribution illustrates a relatively uniform pattern (Figure 4). Notably, significant differences were identified in the seasonal distribution of fractures (*p* < 0.05). Tibial malleolus fractures increased markedly in spring (28.57%, 42), likely due to the rise in physical activity during this time. Meanwhile, trimalleolar fractures peaked in autumn (31.54%, 41).

### 3.5. Mechanism of Injury and Associated Trauma

Out of 1010 fractures analyzed, 33.86% (342) were associated with additional traumas. Trimalleolar and bimalleolar fractures exhibited a higher incidence of dislocations. Peroneal and tibial fractures were more frequently associated with distortive trauma and foot fractures: trimalleolar (*n* = 64) 49.23%, bimalleolar (*n* = 58) 40.28%, tibial malleolus (*n* = 57) 38.78%, and peroneal malleolus (*n* = 163) 27.67%.

Trauma occurred in 31.97% (196 cases) of non-surgically treated fractures. The most common types were distortive trauma (*n* = 108) 55.10%, foot fractures (*n* = 54) 27.55%, dislocation (*n* = 1) 0.51%, and others (*n* = 33) 16.83%.

In cases of bimalleolar fractures, associated trauma was present in 22.58% (7) of instances. The most prevalent complication in these cases was distortive trauma, occurring in 71.43% (5) of instances. For tibial fractures, associated trauma was observed in 40.68% (118) of cases, with 15.25% (18) involving distortive trauma. In the context of peroneal fractures, associated trauma occurred in 37.90% (174) of cases. The predominant complication for non-surgically treated peroneal fractures was distortive trauma, representing 48.85% (85), followed by foot fractures at 22.41% (39). Associated traumas were noted in 36.78% (146) of surgically treated fractures.

In trimalleolar fractures, associated traumas were observed in 51.20% (64) of cases, with dislocation being the most frequent, occurring in 40.77% (53 cases). For bimalleolar fractures, associated traumas were present in 45.13% (51 cases). Notably, surgically treated bimalleolar fractures exhibited a high incidence of dislocations, accounting for 23.40% (33 cases). Surgically managed tibial fractures showed a lower incidence of associated trauma, at 31.03% (9 cases). For peroneal fractures, associated traumas were found in 16.92% (22 cases), with dislocation and distortive trauma being the most common, occurring in 10.77% (14 cases).

### 3.6. Comorbidities

Among the 397 patients who received surgical treatment for ankle fractures, 41.81% (166 patients) had comorbidities. Of these, 34.34% (57 patients) were men, while 65.66% (109 patients) were women (Table 5). A chi-square test was performed to assess the association between comorbidities and both hospital stay duration and treatment modality. Hospital stay was categorized as short (<10 days) or long (≥10 days). No statistically significant association was found between the presence of comorbidities and treatment choice (*p* > 0.05). Similarly, most comorbidities did not show a significant relationship with hospital stay duration. However, a significant association was identified for patients with diabetes, who were more likely to experience hospital stays longer than 10 days (*p* < 0.05).

### 3.7. Treatment

Between 2018 and 2023, a total of 613 fractures were treated non-surgically. The majority were peroneal fractures, accounting for 459 cases (74.88%), followed by 118 tibial fractures (19.25%), 31 bimalleolar fractures (5.06%), and 5 trimalleolar fractures (0.82%). The most prevalent form of conservative management was casting, as detailed in Table 6.

The distribution of treatments included cast splints in 391 cases (63.78%), casting braces in 93 cases (15.16%), cast valves in 76 cases (12.40%), walker braces in 47 cases (7.67%), and bivalve braces in eight cases (1.31%).

A total of 397 fractures were surgically treated, with 45.59% (181) in males and 54.41% (216) in females. The types of fractures treated included 125 trimalleolar fractures (31.49%), 113 bimalleolar fractures (28.46%), 29 tibial fractures (7.3%), and 130 peroneal fractures (32.75%). The most common surgical treatment was an open reduction with internal fixation (ORIF), applied in 316 cases (80.05%). Additional treatment methods included closed reduction with internal fixation (CRIF) in 24 cases (6.05%), external fixation (EF) in 14 cases (3.53%), and external fixation followed by osteosynthesis in 40 cases (10.01%). There was also one instance of arthrodesis (0.25%) and one case of closed reduction without internal fixation (0.25%) (Table 7).

The data regarding the treatment of open fractures reveals that all 19 cases (100%) underwent surgical intervention. According to the Gustilo–Anderson classification, eight cases were classified as Type I, seven as Type II, and four as Type IIIA. Specifically, seven cases were managed with ORIF, accounting for 36.84%, while an equal number were treated with EF (36.84%). The remaining five cases, representing 26.32%, required EF followed by osteosynthesis. No cases of deep postoperative infection were recorded in this subgroup during the hospitalization period.

In the context of trimalleolar fractures, fixation was performed on the posterior malleolus in 30 instances (26.32%), on the medial malleolus in 98 instances (86.84%), and on the lateral malleolus in all 115 cases (100%). Furthermore, 13 cases (11.30%) necessitated the installation of a transsyndesmotic screw.

For bimalleolar fractures, the lateral malleolus was fixed in all 109 cases (100%), while fixation of the medial malleolus was necessary in 69 cases (63.30%). Additionally, 19 cases (17.43%) required the use of a transsyndesmotic screw. Finally, among peroneal fractures, 14 cases (10.85%) required the insertion of a transsyndesmotic screw.

### 3.8. Hospital Stay

The average hospital stay for patients who underwent surgical treatment between 2018 and 2023 was 9.69 ± 6.17 days, with a range of 1 to 56 days. Patients hospitalized for open fractures had an average stay of 18.29 ± 15.20 days, ranging from 5 to 56 days. In contrast, those with closed fractures averaged a stay of 9.29 ± 5.13 days, with a range of 1 to 45 days. Mean hospital stay: trimalleolar 12.62 ± 7.42 (1–56), bimalleolar 9–81 ± 6.30 (2–45), tibial malleolus 8 ± 4.47 (3–24), and peroneal malleolus 6.98 ± 2.90 (1–18).

## 4. Discussion

This study offers a comprehensive evaluation of the epidemiological and clinical characteristics of ankle fractures treated at a tertiary hospital over 6 years. The periods covered include pre-COVID-19 (2018–2019), COVID-19 (2020–2021), and post-COVID-19 (2022–2023). The analysis confirms that the COVID-19 pandemic significantly impacted the incidence of ankle fractures, the management strategies employed, and patient outcomes.

The total number of ankle fractures decreased significantly in 2020, with a 31.70% drop compared to 2019. This finding aligns with international reports indicating a general decrease in orthopedic and trauma cases, attributed to lockdown measures, restrictions on outdoor and sports activities, and the public’s fear of hospital exposure to COVID-19, as reflected in the study by Wong et al., which documented a 41.2% reduction in hospital admission [24]. Notably, the majority of unstable injuries occurred indoors, with 72% indoors compared to 38% outdoors [25,26]. This reduction was particularly pronounced among young individuals in the 10–14 and 25–29 age groups, as well as older adults aged 60–69. These trends reflect the impact of school closures, the shift to remote work, and increased risk aversion among the elderly [27]. The reduction in ankle fractures during 2020 is likely multifactorial. Shelter-in-place mandates and reduced outdoor activities directly limited exposure to traffic, sports, and work-related trauma. Additionally, patient reluctance to seek medical attention during the early pandemic likely contributed to underreporting of low-energy fractures. Age and sex distributions suggest that younger and more active populations experienced the most pronounced decline, while older women continued to sustain fragility fractures indoors.

Following the acute phases of the pandemic, ankle fracture rates gradually recovered during the post-COVID-19 period. However, they did not reach pre-pandemic levels, showing no rebound effect, similar to findings of Stringer et al., who reported rates of 15.20% before the lockdown, 8.81% during the lockdown, and 13.17% after the lockdown [28]. Notably, in 2020, the average age of fracture patients dropped to its lowest, at 45.07 years, according to findings reported by Nath et al. who observed a mean age of 47.73 during the pandemic [29]. This finding contrasts with trends in the literature, which report an increased proportion of elderly patients experiencing fractures during the pandemic due to frailty and domestic accidents [30].

Regarding gender distribution, no significant differences were observed across the three periods, although there was a slight female predominance; these data are consistent with the literature as reported by Mo et al., who recorded an increase of 1.3% (2020) and 1.1% (2021) [31], with 2.4× more female patients compared to male patients reported by Shah et al. [26]. However, trimalleolar fractures demonstrated a significant female bias, particularly among those aged 60–74, consistent with known trends in fragility fractures and a possible osteoporotic etiology [32]. This finding aligns with the literature, which suggests that elderly women are more susceptible to complex ankle fractures due to factors such as reduced bone density, impaired balance, and increased fall risk [1].

Regarding treatment strategies, international guidelines recommended prioritizing conservative management to minimize surgical burden and hospital stays during the pandemic [33]. However, our data show a paradoxical increase in surgical interventions during the COVID-19 period. This trend may indicate a selection bias towards addressing more complex fractures, as simpler cases could have gone unreported or been managed conservatively at home. These findings closely parallel the trends observed by Mascio et al., who reported an increase in surgical interventions from 5.6% to 10.4% during COVID-19, reflecting the severity of the injuries [34]. Additionally, the use of EF rose significantly during COVID-19, probably due to its benefits of reduced operative time and decreased hospital resource demands [35].

Post-pandemic, there was a shift back to pre-COVID-19 surgical preferences, especially regarding ORIF, which rose to account for 81.68% of surgical cases.

During the pandemic, hospital length of stay (LOS) increased and remained elevated after COVID-19, with statistically significant differences observed across the different periods. This increase may be attributed to the higher complexity of fractures and the presence of comorbidities. Although a trend toward longer hospitalizations was observed across the study periods, no statistically significant differences were found between most comorbidities and extended LOS, with the exception of diabetes, which was significantly associated with hospital stays longer than 10 days (*p* < 0.05). This finding suggests that diabetic patients may require more complex perioperative management or longer recovery times. Other cardiovascular risk factors, including hypertension, dyslipidemia, heart disease, and obesity, did not show a statistically significant impact on LOS in this cohort [36].

In non-surgical management, clinicians have increasingly adopted functional braces instead of traditional plaster casts. This shift reflects growing evidence that supports the efficacy and enhanced patient comfort associated with removable braces [37].

The seasonal variation in fracture incidence was statistically significant. Tibial malleolus fractures reached their peak in spring, coinciding with increased outdoor activity. In contrast, trimalleolar fractures peaked in autumn, likely because adverse weather conditions elevated the risk of falls. Similarly, Rydemalm et al. reported that 10–12% of all ankle fractures occurred during the winter months (December to February), highlighting a clear seasonal trend in colder climates [9]. However, it is important to note that the Nordic countries experience long, harsh winters with frequent snow and ice, which significantly increases the risk of slipping-related injuries. In contrast, Italy is characterized by milder winters, with minimal exposure to ice or snow. Therefore, while our data also revealed seasonal peaks, particularly in winter and spring, the underlying mechanisms may differ—potentially reflecting changes in physical activity patterns, indoor falls among older adults, or post-pandemic behavioral shifts rather than direct environmental hazards.

Laterality analysis indicated that more complex fractures, such as the trimalleolar and bimalleolar fractures often linked to ankle instability, were slightly more prevalent on the left side. In contrast, simpler fractures, involving the tibial and peroneal malleolus, were slightly more common on the right side. This pattern may be attributed to limb dominance and the biomechanics of falls, as previously suggested in the literature [38].

Fracture complexity influenced the choice of treatment modality. The majority of trimalleolar and bimalleolar fractures (87%) required surgical fixation, while peroneal and tibial fractures were most often managed conservatively (78%). This correlation aligns with fracture severity and patient age, as older individuals are more likely to undergo surgery due to the complexity of their fractures [39].

Dislocations were most frequently associated with trimalleolar fractures, and nearly all cases required surgical intervention. However, no significant difference was found in hospital stay duration for these patients. Although rare, open fractures were universally treated with surgical fixation. The method of fixation—whether through ORIF, EF, or a staged approach—depended on the severity of the fracture and the extent of soft tissue involvement [40].

This study has several limitations. Its retrospective design and single-center setting may impact the external validity of the findings. However, during the COVID-19 pandemic, the hospital served as the primary referral center for trauma, as most surrounding facilities were converted into COVID-19-dedicated hospitals. This allowed us to collect a consistent and representative dataset during a time of healthcare system reorganization. Moreover, variability in treatment protocols and potential confounding factors were not fully controlled. Treatment decisions were influenced by logistical constraints, particularly during the pandemic, when operating room access and resource availability were limited. Additionally, certain clinical variables—such as time to presentation, adherence to rehabilitation, and detailed functional assessments—were not consistently recorded. This residual heterogeneity limits the ability to draw causal inferences and reinforces the need for future prospective studies with standardized protocols. Moreover, a key limitation was the reliance on univariate tests without multivariate modeling. While descriptive and basic inferential statistics were used, no adjustment was made for confounders such as age, sex, comorbidities, or seasonal variation. Incorporating multivariate regression or time-series analysis in future research could better isolate the independent effects of the COVID-19 pandemic on ankle fracture incidence and management. Finally, the absence of functional outcome measures and long-term follow-up limits the assessment of clinical recovery. The study was designed primarily as an epidemiological analysis to evaluate incidence trends, patient demographics, and treatment approaches across three distinct timeframes: pre-COVID-19, during COVID-19, and post-COVID-19. Future research is needed to investigate the long-term clinical course, functional recovery, and quality of life in patients treated for ankle fractures.

## 5. Conclusions

This study demonstrates how the COVID-19 pandemic significantly changed the epidemiology and management of ankle fractures. It emphasizes the necessity for flexible healthcare strategies that can adapt to epidemiological changes while ensuring quality trauma care, especially for vulnerable populations like elderly women.

## Figures and Tables

**Table 1:** Number of fractures divided by period (Pre-COVID-19, COVID-19, and Post-COVID-19) and year.

Period	Total	Year	Total
Pre-COVID-19	396	2018	172
		2019	224
COVID-19	302	2020	153
		2021	149
Post-COVID-19	312	2022	168
		2023	144
Total	1010	Total	1010

**Table 2:** Annual incidence of ankle fractures per 100,000 population/year during 2018–2023 in Catania. Population data were obtained from Italian government statistics.

Population					
Year	Total		Male		Female
2018	311.620		150.037		161.583
2019	297.752		142.792		154.960
2020	296.266		142.230		154.036
2021	300.356		143.635		156.721
2022	301.104		144.999		156.105
2023	299.730		144.256		155.474
**Incidence**					
**Total**		**Male**		**Female**	
55		63		48	
75		69		77	
52		46		56	
50		44		55	
56		59		53	
48		44		51	

**Table 3:** Annual fracture distribution by gender and male–female ratio.

Fractures				Ratio
Year	Total	Male	Female	M:F
2018	172	94	78	1.21:1
2019	224	104	120	1:1.15
2020	153	66	87	1:1.34
2021	149	63	86	1:1.37
2022	168	85	83	1.02:1
2023	144	64	80	1:1.25
Total	1010	476	534	1:1.14

**Table 4:** Number of patients with ankle fractures divided by type of malleolus involved. Distribution by gender and mean age of male and female.

Number				Mean Age	
Type of Fracture	M	F	M:F	M	F
Trimalleolar	36	94	1:2.61	48.96 ± 19.34 (16–83)	60.21 ± 15.36 (20–90)
Bimalleolar	65	79	1:1.20	46.70 ± 19.34 (16–92)	60.60 ± 19.12 (17–93)
Tibial malleolus	95	52	1.93:1	40.00 ± 17.78 (11–78)	44.40 ± 20.86 (5–77)
Peroneal malleolus	280	309	1:1.12	41.48 ± 20.21 (6–89)	46.50 ± 19.85 (4–89)

**Table 5:** Number and percentage of comorbidities occurring in surgically treated fractures divided by gender (others include Chron’s disease, eye disease, celiac disease, gastritis, and Lupus).

Total				Percentage		
Comorbidities		M	F		M	F
Hypertension	89	34	55	53.61%	38.20%	61.80%
Diabetes	31	14	17	18.67%	45.16%	54.84%
Cardiological disease	26	11	15	15.66%	42.31%	57.69%
Dyslipidemia	22	8	14	13.25%	36.36%	63.64%
Psychiatric disorders	14	3	11	8.43%	21.43%	78.57%
Urological disorders	10	5	5	6.02%	50.00%	50.00%
Hematological disorders	9	2	7	5.42%	22.22%	77.78%
Respiratory disorders	9	4	5	5.42%	44.44%	55.56%
Obesity	8	1	7	4.82%	12.50%	87.50%
Oncology	8	1	7	4.82%	12.50%	87.50%
Thyroid disorders	7	0	7	4.22%	0%	100%
Neurological disorders	7	2	5	4.22%	28.57%	71.43%
Dermatological disorders	4	0	4	2.41%	0%	100%
Hepatic disorders	3	2	1	1.81%	66.67%	33.33%
Others	13	6	7	7.83%	46.15%	53.58%

**Table 6:** Ankle fractures classified by conservative treatment: number of fractures and percentage.

	Treatment					Percentage				
Type of Fracture	Splint	Cast Brace	Cast Valve	Bivalve Brace	Walker Brace	Splint	Cast Brace	Cast Valve	Bivalve Brace	Walker Brace
Trimalleolar	5	0	0	0	0	100%	0%	0%	0%	0%
Bimalleolar	19	77	0	0	5	18.81%	76.24%	0%	0%	4.95%
Tibial malleolus	62	21	17	3	15	52.54%	17.80%	14.41%	2.54%	12.71%
Peroneal malleolus	305	63	53	5	33	66.45%	13.73%	11.55%	1.09%	7.19%

**Table 7:** Types of ankle fractures classified by surgical treatment: number of fractures and percentage. Legend: ORIF (open reduction with internal fixation); CRIF (closed reduction with internal fixation); EF (external fixation); EF + ORIF (external fixation with subsequent ORIF); A (arthrodesis); CR (closed reduction without internal fixation).

	Treatment						Percentage					
Type of Fracture	ORIF	IRIF	EF	EF + O	Arthrodesis	CR	ORIF	IRIF	EF	EF + O	Arthrodesis	CR
Trimalleolar	78	5	9	32	1	0	62.40%	4%	7.20%	25.60%	0.80%	0%
Bimalleolar	93	8	5	8	0	0	81.42%	7.08%	4.24%	6.40%	0%	0%
Tibial malleolus	22	6	0	0	0	1	75.86%	20.69%	0%	0%	0%	3.45%
Peroneal malleolus	124	5	1	0	0	0	95.38%	3.85%	0.77%	0%	0%	0%

## Data Availability

Data is contained within the article.
